# Phenotypic and Genotypic Features of a Chinese Cohort with Retinal Hemangioblastoma

**DOI:** 10.3390/genes15091192

**Published:** 2024-09-11

**Authors:** Liqin Gao, Feng Zhang, J. Fielding Hejtmancik, Xiaodong Jiao, Liyun Jia, Xiaoyan Peng, Kai Ma, Qian Li

**Affiliations:** 1 Beijing Tongren Eye Center, Beijing Tongren Hospital, Capital Medical University, Beijing Key Laboratory of Ophthalmology and Visual Sciences, No.1 Dongjiaominxiang, Beijing 100730, China; gaolqin@126.com (L.G.); trdrzhangfeng@163.com (F.Z.); jialiyun@126.com (L.J.); 74000041@ccmu.edu.cn (X.P.); mkpacer@gmail.com (K.M.); 2 Ophthalmic Molecular Genetics Section, Ophthalmic Genetics and Visual Function Branch, National Eye Institute, National Institutes of Health, Rockville, MD 20892, USA; hejtmancikj@nei.nih.gov (J.F.H.); jiaox@nei.nih.gov (X.J.)

**Keywords:** retinal hemangioblastoma, Von Hippel–Lindau gene (*VHL*), genotype, phenotype, large genomic deletion

## Abstract

**Purpose:** To delineate the genotype and phenotype of RH in a Chinese cohort. **Methods:** A group of 51 Chinese probands with RH across 76 eyes was assembled and underwent complete retinal imaging examinations. Sanger sequencing and universal primer quantitative fluorescent multiplex–polymerase chain reaction (UPQFM-PCR) were employed for mutation detection in the coding region of the Von Hippel–Lindal (*VHL*) gene. For frequency calculation, our series was combined with three large cohorts of East Asian descent through a literature review. **Results:** The Von Hippel–Lindal (VHL) syndrome was excluded in fifteen patients (median age: 32.00 years) with unilateral solitary RH. Thirty-six patients of younger ages (median: 22.00 years, *p* = 0.008, Mann–Whitney test) conformed to the diagnostic criteria of the VHL syndrome, and thirty-four patients were genetically confirmed. There were four novel variants identified in the *VHL* gene. Codons 167, 161 and 86 exhibited a mutation occurrence of more than 5% after pooling with literature data, and the large genomic deletion demonstrated a frequency of 17.65%. The RHs were classified as “extrapapillary”, “juxtapapillary” and “mixed” types in 53, 7 and 5 eyes, respectively. Almost all extrapapillary RH lesions were found in the peripheral retina. Hemangioblastomas in the central nervous system (CNS) were observed in 25 out of 31 kindreds (80.65%) with full systemic evaluation data. **Conclusions:** VHL-associated RH might exhibit earlier onset than non-VHL RH. Large genomic deletions were observed at a notably high frequency in the Chinese series with VHL-associated RH, which might be associated with East Asian ethnicity background. RH could potentially serve as an early indicator of CNS hemangioblastoma.

## 1. Introduction

Retinal hemangioblastoma (RH) is a benign tumor demonstrating vascular attributes, originating either from the neurosensory retina or the optic disc [1]. If unmanaged, RH can considerably impair vision or even cause blindness, substantially affecting the active working population. RH tends to occur either sporadically or as a subset of central nervous system (CNS) hemangioblastoma [2,3,4,5,6,7,8,9,10,11] in the Von Hippel–Lindau (VHL) syndrome (OMIM #193300) [12,13,14,15], an inherited autosomal dominant disorder caused by mutations in the VHL tumor suppressor gene (*VHL,* OMIM#608537). *VHL* mutant heterozygotes are predisposed to multiple tumors, including cerebellar, spinal and retinal hemangioblastomas, renal cell carcinomas, pheochromocytomas and lymphatic sac tumors. Therefore, timely identification, precise detection and immediate intervention are vital for efficient management of such condition.

The *VHL* gene comprises three exons and encodes a 213-amino-acid protein with a predicted molecular mass of 24 kD. VHL transcripts show multiple variants, including the use of an alternate in-frame initiator methionine at position 54 and two splice variants having the same first but alternate or missing second exons. VHL regulates the hypoxia-inducible factor (HIF) by acting as a ubiquitin ligase as part of the VCB–CUL2 complex with elongin B, elongin C and Cullin 2 (CUL2). Mutations of the *VHL* can impair its tumor- suppressive function directly or indirectly through HIF-mediated effects. HIF contributes to glucose uptake and the transcription of genes involving growth, mitosis and angiogenesis, such as transforming growth factor α, platelet-derived growth factor, vascular endothelial growth factor and erythropoietin—factors, which have also been implicated in tumorigenesis [14,15,16].

The correlations between germline *VHL* mutations and disease phenotypes have shown inconsistency across ethnic backgrounds [4,5,8,9,10,11,17], stressing the need to characterize this disease’s genotype and phenotype traits across populations. Currently, the information on Chinese patients diagnosed with RHs remains scarce. This study employs Sanger sequencing and universal primer quantitative fluorescent multiplex–polymerase chain reaction (UPQFM-PCR) to screen germline mutations of *VHL* in 51 unrelated Chinese probands diagnosed as RH, potentially enabling effective outlining of the clinical and genetic features of this disease in the Chinese population. 

## 2. Methods

### 2.1. Study Subjects

Institutional Review Board (IRB) approval was obtained from the Ethics Committee of the Beijing Tongren Hospital, Capital Medical University, Beijing, China, and the current study adhered to the tenets of the Declaration of Helsinki. Written informed consent was obtained from each patient. 

### 2.2. Clinical Evaluation

A thorough retrospective evaluation of medical records from all probands and their family members, when available, was conducted. This included ophthalmic examinations and retinal imaging, incorporating fundus photography and fluorescein angiography, interpreted independently by two retinal specialists (LG and QL). The RHs were categorized as juxtapapillary and extrapapillary, as described previously [11,12]. In each eye affected, the diameter of the largest RH was measured. To assess potential systemic involvement in the VHL syndrome, cerebral imaging and abdominal ultrasonography data from each patient’s medical record were reviewed, and family histories were collected by interviewing the probands and family members if available. The diagnostic criteria for the VHL syndrome adhered to international, Danish or Dutch guidelines [18,19,20], stipulating that either one hemangioma accompanied by a positive family history or at least two hemangiomas or one hemangioma coupled with a visceral lesion without a positive family history were necessary for clinical diagnosis. Alternatively, molecular diagnoses could be set by identifying a heterozygous pathogenic (or likely pathogenic) variant in *VHL* through molecular genetic testing [14].

### 2.3. VHL Gene Screening

All patients had their peripheral blood collected in a sample for germline mutation screening of the *VHL* gene. Genomic DNA was extracted; polymerase chain reaction (PCR) amplification was utilized; and Sanger sequencing was performed. Universal primer quantitative fluorescent multiplex–polymerase chain reaction (UPQFM-PCR) was performed to detect the large deletions and confirmed via real-time quantitative PCR, as described previously [21].

### 2.4. In Silico Analysis

Variants were evaluated in terms of novelty and pathogenicity by reviewing publicly available databases, including gnomAD [22], ClinVar, Franklin (https://franklin.genoox.com/clinical-db/home, 12 July 2023) and HGMD [23], and for in silico pathogenicity predictions, Polyphen2 [24] and Mutation Taster were used [25]. Variants were also evaluated in accordance with American College of Medical Genetics and Genomics–American Association of Molecular Pathology (ACMG-AMP) 2015 sequence [26] or 2020 ACMG-ClinGen CNV guidelines [27]. 

### 2.5. Literature Review and Data Combination for Mutation Frequency Calculation

In order to enhance the precision of mutant frequency calculation, which may have been influenced by our relatively small sample size, we also conducted a literature review [10,28,29] using Pubmed and Googlescholar, resulting in a total of 203 VHL syndrome patients affected by RH. We extracted the following data: (i) genetic information—sequence variation at the DNA and protein levels and type of variation (e.g., missense); (ii) clinical information—diagnosis of central nervous system hemangioblastoma and retinal capillary hemangioblastoma (RCH); (iii) patient race and familial or sporadic inheritance. Subsequently, the frequency was calculated. These analyses were adjusted for familial relationships.

## 3. Results

### 3.1. Demographic Features of the RH Cohort (Table 1)

A total of 51 unrelated Chinese probands, comprising 26 with a positive family history and 25 sporadic cases (25 males and 26 females), were recruited. These probands represented 76 eyes with RHs diagnosed at the Beijing Tongren Eye Center. At the time of their first visit, the median age was 24 years (range: 8–58 years). The median follow-up duration for this cohort spanned 22 months (range: 0–264 months), while only baseline medical records were available for 14 eyes of 7 patients. The VHL syndrome was excluded via *VHL* gene sequencing in fifteen patients with a median age of 32 years (range: 13–51 years), who only exhibited unilateral solitary RH and had a negative family history. In contrast, 36 patients with a significantly lower median age of 22 years (range: 8–58 years, *p* = 0.008, Mann–Whitney test) met the clinical and genetic diagnostic criteria for the VHL syndrome. Among these 36 VHL syndrome cases, 21 showed bilateral RHs at their initial visit. Over the follow-up duration of 17–87 months (median: 46 months), three cases initially shown as unilateral RHs presented new RH development in the unaffected eye, resulting in 24 instances of bilateral occurrence (66.67%). Comprehensive medical record data regarding cerebral and abdominal involvements were available in 31 out of the 36 VHL syndrome kindreds, with 25 out of these 31 kindreds exhibiting hemangioblastomas in the central nervous system (CNS, 80.65%), 7 with renal cell carcinoma (22.58%), 5 with pancreatic cysts (16.13%) and 2 with pheochromocytoma (6.45%). A pedigree presentation of patients with a positive family history of VHL is shown in the Supplemental Data Files. 

**Table 1:** Summary of phenotypic and genotypic data.

Nbr	Gender	Age	*VHL* Genotypes	Kindred Phenotypes	Affected Eyes	Systemic Phenotypes		Clinical VHL
						CNS	Abdominal Organs	
P1	F	19	ND	N	RE	N	N	N
P2	F	47	ND	N	RE	N	N	N
P3	M	35	ND	N	RE	N	N	N
P4	M	20	ND	N	RE	N	N	N
P5	F	34	ND	N	LE	N	N	N
P6	M	40	ND	N	RE	N	N	N
P7	F	32	ND	N	LE	N	N	N
P8	M	51	ND	N	RE	N	N	N
P9	F	22	ND	N	LE	N	N	N
P10	F	45	ND	N	RE	N	N	N
P11	M	19	ND	N	RE	N	N	N
P12	M	21	ND	N	RE	N	N	N
P13	M	40	ND	N	LE	N	N	N
P14	M	43	ND	N	LE	N	N	N
P15	F	36	ND	N	RE	NA	NA	N
P16	F	8	ND	Y,C	BE	N	N	Y
P17	F	57	ND	Y,C	BE	N	N	Y
P18	M	22	p.Ser65Leu	Y,C	LE	N	N	Y
P19	M	22	p.Ser65Trp	Y,C	RE	N	Pancreatic cysts	Y
P20	M	13	p.Ser65Trp	N	RE	N	NA	N
P21	M	51	p.Glu70Lys	N	BE	N	N	Y
P22	F	11	p.Asn78IIe	Y,C	BE	N	N	Y
P23	F	40	p.Asn78Ser	Y,C	RE	N	N	Y
P24	F	28	p.Arg79Pro	N	BE	Y	N	Y
P25	M	25	p.Pro86Ser	Y,C,R,K	BE	Y	N	Y
P26	M	14	p.Leu85Leufs*46	Y,C	LE	NA	NA	Y
P27	F	25	p.Pro86Thr	N	RE	NA	NA	N
P28	F	16	p.Leu89Pro	Y,C,R	BE	N	N	Y
P29	F	9	p.Gly93Asp	N	BE	N	N	Y
P30	F	13	p.Tyr98*	Y,C,K	LE	Y	N	Y
P31	M	30	p.His110fs*49	Y,C,R	BE	Y	N	Y
P32	F	58	p.Tyr112Ser	Y,C	RE	Y	Liver, kidney cysts	Y
P33	M	18	p.Trp117Gly	Y,C,R,K	BE	Y	NA	Y
P34	F	29	p.Asn131Lys	N	RE	N	NA	N
P35	F	19	p.Thr157IIe	Y,C,R,K	BE	N	N	Y
P36	F	22	p.Lys159Glu	Y,C	BE	N	NA	Y
P37	F	23	p.Arg161*	Y,C,R	BE	Y	Pancreatic cysts	Y
P38	M	22	p.Arg161*	N	BE	Y	Pancreatic cysts	Y
P39	M	15	p.Arg167Gln	N	BE	N	N	Y
P40	F	32	p.Arg167Trp	Y,C,R	BE	N	N	Y
P41	F	13	p.Arg167Trp	Y,C	BE	Y	N	Y
P42	M	48	p.Arg167Trp	Y,C,R	BE	N	Pheochromocytoma	Y
P43	F	24	p.Pro172Argfs*30	Y,R	BE	Y	N	Y
P44	M	35	p.176delArg	Y,C	RE	N	N	Y
P45	F	32	c.463+3 A>G	Y,R	BE	Y	Pheochromocytoma	Y
P46	M	20	deletion of Exon 1	Y,C,R,K	BE	Y	Pancreatic cysts	Y
P47	M	38	deletion of Exon 1	Y,C,R,P	BE	N	Kidney carcinoma, pancreatic cysts	Y
P48	M	17	deletion of Exon 2	Y,C,R	BE	Y	N	Y
P49	M	16	deletion of Exon 2, 3	Y,C,R	BE	Y	N	Y
P50	M	24	deletion of Exon 2, 3	Y,C,R	BE	Y	N	Y
P51	F	23	deletion of Exon 2, 3	NA	RE	NA	NA	NA

F: female; M: male; ND: not detected; N: no; Y: yes; NA: not available; RE: right eye; LE: left eye; BE: both eyes; CNS/C: central nervous system; R: retina; K: kidney; P: pancreatic changes.

A total of 28 germline variants were identified in the *VHL* gene (Table 1 and Table 2) in 34 cases affected by RH, of which 30 (88.23%) satisfied the clinical criteria for the VHL syndrome. Four cases (P20, P27, P34 and P51) exhibiting a solitary unilateral RH without evidence of systemic involvement or family lineage were found to harbor the deleterious variants p.Ser65Trp [30], p.Pro86Thr [31], p.Asn131Lys [10] and a deletion of Exon 2, 3 of the *VHL* gene, respectively. In 17 individuals, no pathogenic changes were identified, although 2 of them (P16 and P17) were clinically diagnosed as having the VHL syndrome.

### 3.2. Genotype

**Figure 1:**
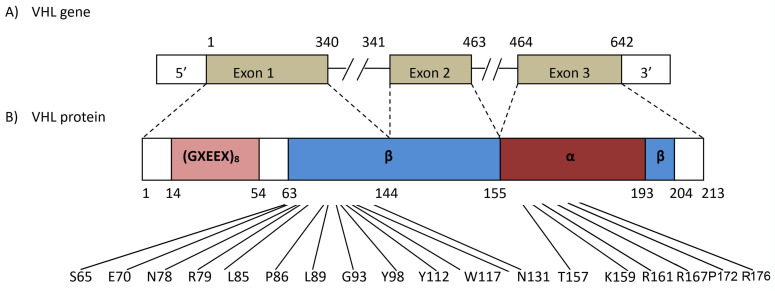
The corresponding position of the missense mutants in the current study. The *VHL* gene comprises three exons and encodes a 213-amino-acid protein. The VHL gene has two domains: the β domain and the α domain. The β domain is roughly 100 residues long and is rich in the β sheet. The α domain is smaller and α-helical. The two domains are held together by two linkers and a polar interface.

**Figure 2:**
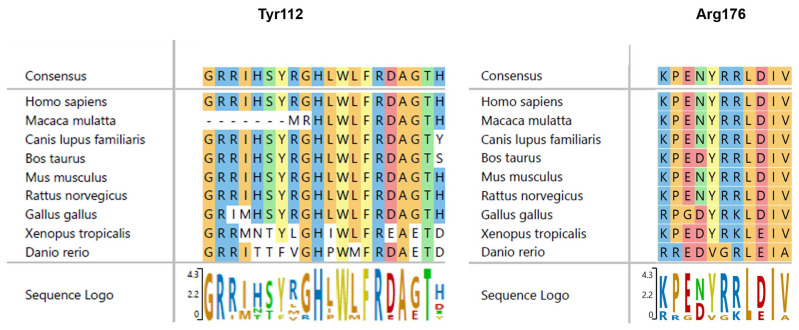
Protein sequence alignment of human VHL protein with VHL homologs from other species. The mutated residues are highly conserved during evolution.

Of the 28 potentially disease-causing variants of the *VHL* gene (Table 2), 4 were novel, including an SNV (c.335A>C, p.Tyr112Ser), an in-frame deletion (c.528_530delGAG, p.176delArg), an insertion with a resulting frameshift and premature termination (c.254insT, p.Leu85Leufs*46) and an indel with a resulting frameshift and premature termination (c.515delCinsGCT, p.Pro171Argfs*30), none of which were found in the HGMD, ExAC or 1000G databases. An additional 19 previously reported variants were identified, as well as 4 large deletions including exons 1, 2, and 2 and 3 identified by UPQFM-PCR. Figure 1 and Figure 2 and Table 2 summarize the characteristics of these mutations.

**Figure 3:**
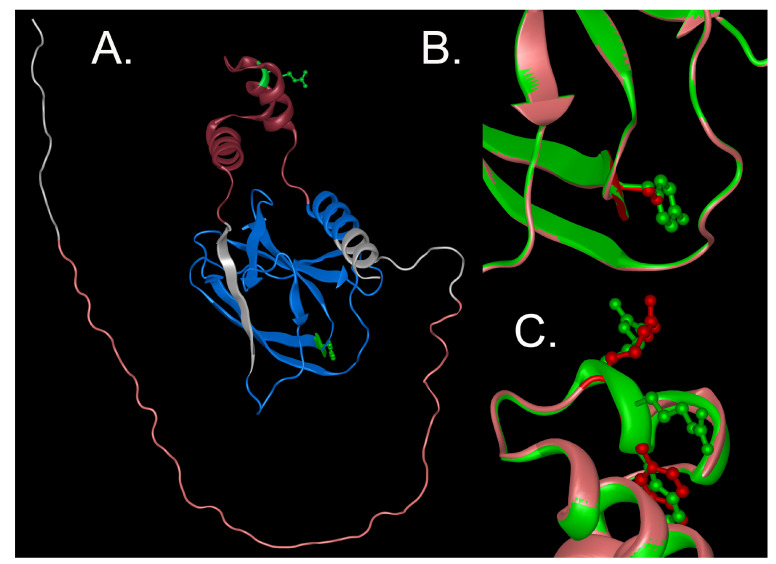
Structure of the VHL protein. **A**. An overview of the VHL protein structure showing the GXEEX repeats in pink, the beta domain in blue and the alpha domain in red, as well as the locations of the p.Tyr112Ser mutation in the beta domain and the p.176delArg mutation in the alpha domain. **B**. An enlarged view of the p.Tyr112Ser mutation with the wild type shown in red, with the tyrosine shown in ball and stick format, and the ser112 mutant protein shown in red, with the serine in ball and stick format. Little or no change is seen in the backbone protein fold. **C**. An enlarged view of the p.176delArg mutation with the wild-type protein shown in green and the p.176delArg mutant shown in red. The protein backbone fold is conserved, and changes in the positions of the adjacent tyr175 and arg177 residues are minimal.

The novel c.528-530del GAG mutation (p.176delArg) in exon 3 is an in-frame deletion of an Arginine residue residing between val175 and ser177 in the middle of the α-domain of VHL (Figure 3). The Arg176 deletion does not impact the protein fold greatly but does lie in a critical region for VHL function. 

The frameshift mutation c.254insT (p.Leu85Leufs*46) in exon 1 is located in the β-domain, introducing a frameshift after codon 85 and a premature termination at codon 130, substituting random sequence for most of the β-domain and eliminating part of the β- and all of the α-domains. The c.515delCinsGCT frameshift is located in the α-domain, substituting random sequence for the carboxyl part of the α-domain and the distal β-domain and loss of the last 11 amino acids. Both would be predicted to have severe effects on the VHL structure.

The novel missense p.Tyr112Ser mutation was predicted to be probably damaging by Polyphen 2 and deleterious by Mutation Taster and yielded an ALIGN score of 143.11. It fit the ACMG criteria PM1, PP2, PM2, PM5, PP3, PP5. 

Of the 213 codons that constitute the coding sequence of VHL, codon 167 was found to be the most frequently mutated and was observed in approximately 11.76% of the 34 genetically confirmed participants. This was followed by codons 65 (8.82%), 161, 86 and 78 (5.88% each). No individual or pedigree was found to carry the mutation in more than one codon. There were six cases where large deletions, including deletions of exon1, exon2, as well as exons 2 and 3, were detected, accounting for 17.65% of the cases (Table S1). 

Codons 167, 65, 161, 86 and 78 were observed to have the highest frequency of mutations in the current cohort of individuals of Chinese descent. After data combination with literature data, codons 167, 161 and 86 exhibited a mutation occurrence greater than 5%, while codons 65 and 78 demonstrated a decreased mutation frequency after the combination. The deletion of exon(s) demonstrated a frequency of 17.65% in the current investigation and 20.99% when combined with the data obtained from previous studies (Table S1). 

### 3.3. Ocular Phenotype 

The baseline best corrected visual acuity (BCVA) in logMAR for the RH-affected eyes recruited in this study (*n* = 76, 51 cases) was 0.50 (equating to 20/63 in Snellen VA, range: −0.20–3.00). Upon the initial consultation, severe visual loss, defined as Snellen VA less than 20/200, was identified in 20 eyes (26.32%), while monocular blindness (<20/400) was observed in 14 eyes (18.42%). At their final appointments (62 eyes of 44 cases), the median BCVA in logMAR for these RH-affected eyes was 1.00 (20/200 in Snellen VA, range: −0.19–3.00). Severe visual loss was noted in 31 eyes (50.00%), and BCVA lower than 20/400 was observed in 23 eyes (37.10%). Moreover, by the time of these concluding visits, nine cases (20.45%) had progressed into bilateral blindness.

At baseline examination of the affected eyes, except for the 11 eyes (11 patients) with phthisical changes or visual axis opacification, which preclude fundus observation, the RHs were identifiable under ophthalmoscope in 65 eyes of the 41 cases subjected to evaluation of the RHs. As defined by the location of the RHs [11], they were grouped into three categories: the “extrapapillary” type evident in 53 eyes (81.54%) from 31 cases, the “juxtapapillary” type in 7 eyes (10.77%) from 7 cases and the “mixed” type, observable simultaneously in both the retina and the optic nerve, found in 5 eyes (7.69%) from 5 cases. However, no significant variations were found in the BCVA (logMAR) between these three types. 

**Figure 4:**
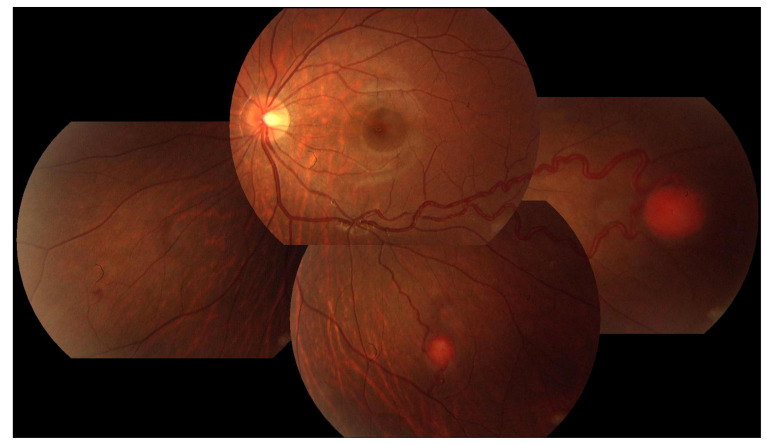
**Color fundus photography (CFP) of the left eye of case P28.** Typical multiple extrapapillary RH in the peripheral retina outside the vascular arcade.

**Figure 5:**
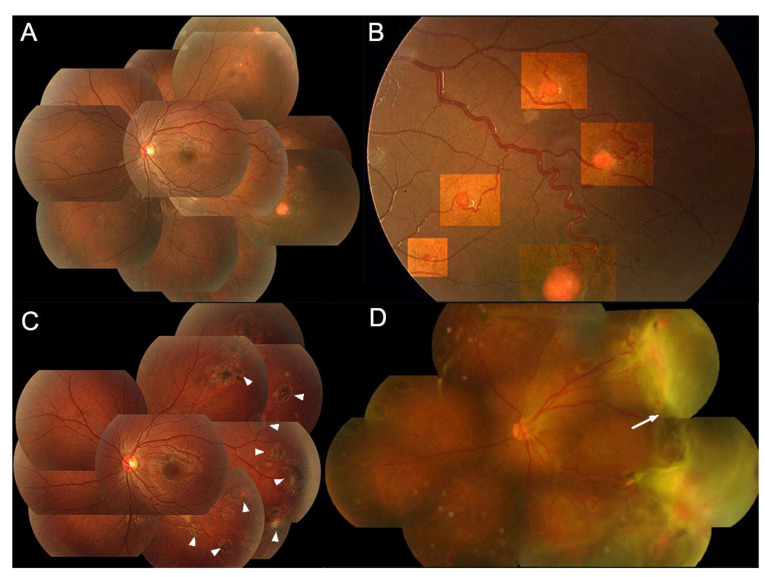
Color fundus photography (CFP) of the left eye of case P49 illustrates both the therapeutic efficacy of laser ablation for retinal hemangioblastomas (RHs) and the progression of retinopathy over a 7-year period without ocular examination or treatment. (**A**,**B**): Multiple typical extrapapillary RHs in the peripheral retina outside the vascular arcade, highlighted in (**B**). (**C**): Shrinkage of the RHs one year after laser ablation treatment (arrowheads). (**D**): The patient did not return for ocular examination until 7 years later, presenting with extensive exudative retinal detachment in the temporal region (arrow).

**Figure 6:**
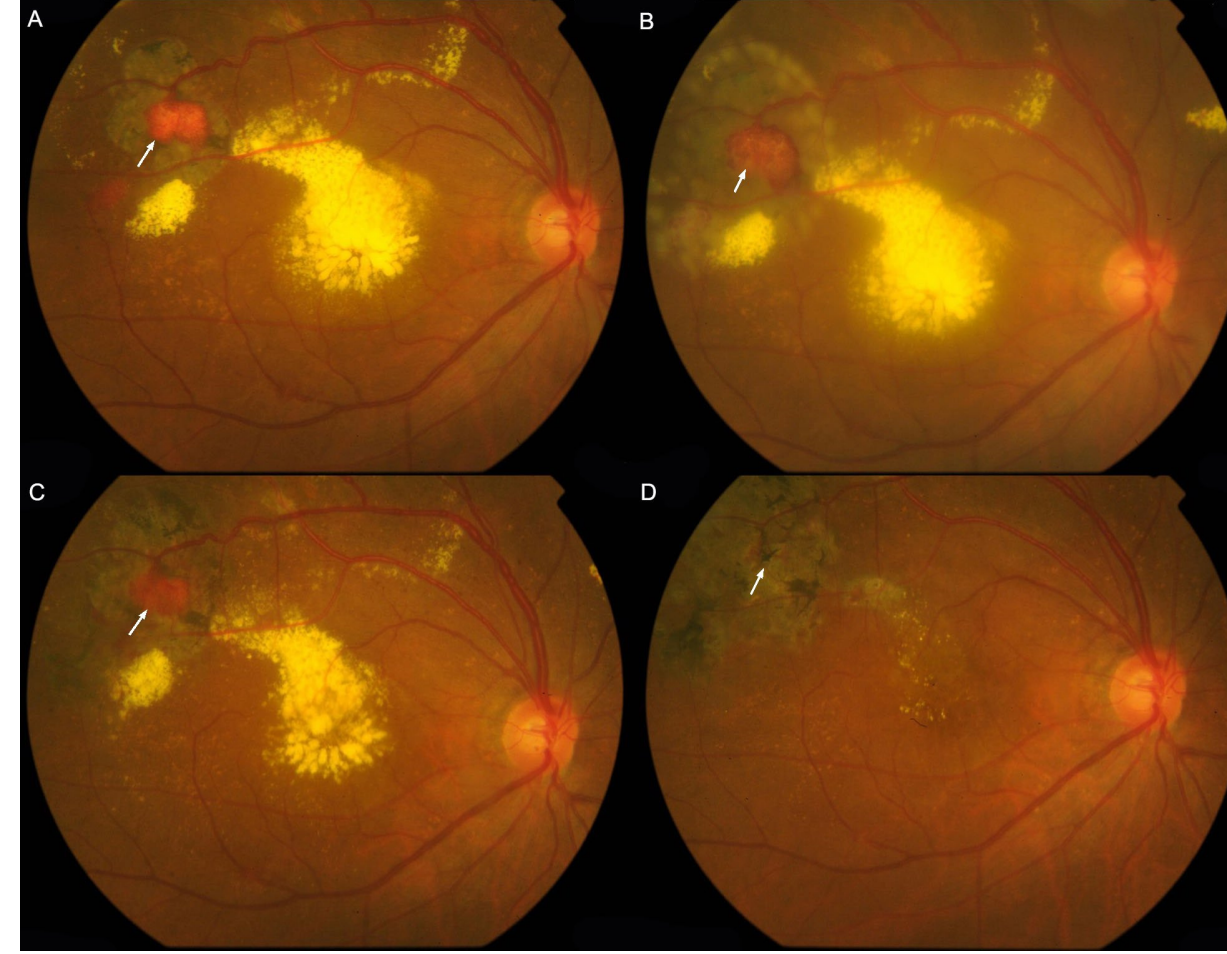
Color fundus photography (CFP) of the right eye of case P32 illustrates the therapeutic efficacy of RH laser ablation. Shrinkage of the RHs (arrows) is evident, along with resolution of exudates in the macula following RH ablation. (**A**), The patient’s initial visit in our institution showed RH near the vascular arcade surrounded by laser spots. (**B**,**C**), After multiple laser treatments, the RH appeared in smaller size and less exudates in the macula. (**D**), The exudates in macula almost resolved.

For the treatment-naïve eyes with extrapapillary RHs (n = 53), virtually all the lesions, except the right eye of P35, were in the peripheral retina outside the vascular arcade (Figure 4 and Figure 5A,B), primarily beyond the equator. The most frequent ocular complications encountered were exudative retinal detachments, discernible in 17 eyes (32.08%). Table 3 details the complications, treatments used and visual outcomes at the concluding exams of the extrapapillary RHs. The occurrence of exudative retinal detachment (ERD) is notably elevated in eyes with RHs larger than 4.5 mm (77.78%). Concurrently, the incidence of baseline macular involvement is lowest (0%) in eyes with an RH diameter of ≤1.5 mm while being statistically significantly higher in eyes with RHs larger than 4.5 mm (88.89%) and RHs ranging from 1.5 mm to 4.5 mm (58.33%, *p* < 0.001, Table 3). Regarding treatments, RHs with a diameter of ≤1.5 mm showed a notable response to laser therapy, leading to regression of both the RHs and associated exudates (Figure 5C and Figure 6A–D). However, the long-term efficacy of treatments largely depends on the growth rate of existing and newly formed RHs, as well as patient compliance with follow-up and management (Figure 5D).

For the treatment-naïve eyes with solitary juxtapapillary RHs (*n* = 7), tumors were commonly observed emerging from the disc margin or the surface. During the disease progression, macular involvement was observed in almost all patients as exudates, ERD in macula and macular edema, inducing early central vision loss.

### 3.4. Phenotype–Genotype Analysis

The observed incidence rates of bilateral RHs in patients with point mutations (missense and in-frame deletion), truncated mutations (nonsense, frameshift and splicing) and large genomic deletion were 70.00%, 83.33% and 83.33%, respectively. Although no statistical significance was determined, the occurrence of bilateral involvement seemed more prevalent in patients with null mutations (splicing, exon deletions, nonsense and frameshift) compared with those with point mutations. Analogously, consideration was given to the presence of multi-system involvement within each proband’s family (affecting organs beyond the eyes). Multi-system involvement was identified in 75% of kindreds bearing point mutations, while it was found in all pedigrees with null mutations, manifesting as cerebral hemangioblastomas, renal cysts, renal cell carcinoma (RCC), pheochromocytoma, pancreatic cysts and epididymal cysts. Pheochromocytoma was identified in two distinct probands (P42 and P45), with each harboring variants c.463+3 A>G and p.Arg167Trp, respectively.

## 4. Discussion

### 4.1. Genotypic Features

Codons 167, 161 and 86 in the current cohort of individuals of Chinese descent exhibited a mutation occurrence greater than 5%, even pooling with data from previous literature (Table S1). In a sizable cohort by Mettu et al. [8] consisting mostly of individuals of Caucasian descent affected by VHL-associated RH (pedigree n = 135), codons 167, 78, 65 and 161 were identified as mutation hotspots, indicating that codons 167 and 161 are common hotspots across ethnicities. This observation is not unexpected, as these two hotspots were also the primary codons affected by mutations in the overall VHL syndrome population, regardless of the presence of RH [10,29].

Large deletions that result in failure of protein production were observed at a notably high frequency of 17.65% in our series of RHs, close to the frequencies found in data based on East Asian cohorts (22.73% [29] and 19.51% [10]). This frequency is significantly higher than the 2.69% reported by Wong et al. [11] in a large cohort of VHL syndrome patients, primarily comprising white individuals with RH complications. Although Wong et al. [11] demonstrated that complete absence of VHL from an allele results in less frequent eye disease than a focally mutated or truncated protein, our current study observed a higher prevalence of bilateral involvement in patients with exon deletions (83.33%) than in those with point mutations (70%), although this difference did not reach statistical significance. Wong et al. have proposed that the occurrence of RH may involve adjacent sequences flanking *VHL* or compensatory mechanisms in the retina [11]. However, we assume that the low occurrence of RH observed by Wong and colleagues [11] is not primarily related to genotypic or molecular issues but rather to the low prevalence of the mutant in the specific population, since a substantial racial disparity in the distribution of this specific type of mutation within the overall VHL syndrome population was shown (Tamura et al. established a prevalence of 23.43% [29] and Wong et al. of 32.47% [10] in Asians; this compares to a prevalence of 7.43% observed by Wong et al. [11] in patients of predominantly white ethnicity and a prevalence of 11% ascertained by Nordstrom-O’Brien et al. in north European patients [32]). 

Large genomic deletions have been shown to involve genes neighboring the *VHL* gene that can potentially impact the manifestation of the disease. For instance, large deletions of *VHL* and C3orf10 [17], as well as BRK1, have been associated with lower lifetime risk of kidney cancer [33], whereas the deletion encompassing *VHL* and the FANCD2 gene may be linked to an increased susceptibility to breast cancer [34]. Since all of the genetic diagnoses in our series were completed a decade ago, Sanger sequencing and UPQFM-PCR were the major sequencing strategies used in the current study, focusing on specific exons of interest. Nowadays, next-generation sequencing (NGS) has experienced extensive utilization in the genetic diagnosis of the VHL syndrome due to its broader coverage and enhanced efficiency. Given the high prevalence of exon deletions in the *VHL* gene among the East Asian population, we propose that extra attention should be given to the proximity of the *VHL* gene when interpreting cases with large genomic deletions for potential risk or favorable prognosis associated with each case.

### 4.2. Phenotypic Features

In our study, we found that cases clinically or genetically diagnosed as the VHL syndrome demonstrated a median age of 22 years at the initial consultation, significantly younger than the 32 years observed in patients with unilateral solitary RHs. This correlates with the scenario of CNS hemangioblastomas, wherein VHL-associated tumors typically present about two decades earlier than sporadic tumors [13]. This differentiation is in line with the one-hit and two-hit model of tumorigenesis (as seen in retinoblastoma). Such distinction observed among individuals, caused by somatic and germline mutations, is unsurprising, and it is consistent with the one-hit and two-hit model of tumorigenesis (as in retinoblastoma) [7]; germline mutations, or initial somatic mutations present in the zygote, are carried forward to all adult cells, resulting in an earlier manifestation of the disease [35]. 

CNS hemangioblastoma was observed to coexist in 80.65% of our pedigrees with VHL-associated RH and in a higher percentage compared to RCC (22.58%), pancreatic cysts (16.13%) and pheochromocytoma (6.45%). The two cohorts from East Asia and one from Iran also showed a similarly high coexistent percentage of CNS hemangioblastoma (Wong et al.: 81.39% [10]; Tamura et al.: 73.20% [29]; and Naseripour et al.: 58.82% [9]). Such close association between hemangioblastoma in the CNS and the retina is further supported by a murine model of VHL-associated RH conducted by Wang et al. [36,37]. The study revealed dilated vasculature in the brainstems of all the mice showing RHs, while no specific lesions were found in the kidney or pancreas, suggesting that embryonically derived hemangioblasts may be the common origin of both VHL-associated retinal and CNS hemangioblastomas. RH has been reported as the initial manifestation of the VHL syndrome in 43% to more than 50% of patients [37,38]. Therefore, we propose that RH could potentially serve as an early indicator of CNS hemangioblastoma, and once detected, immediate systemic evaluation and close surveillance should be pursued.

The vast majority of extrapapillary RHs observed in this study were found in the peripheral retina at the baseline visit rather than in the posterior retina. This predilection for locations is supported by pathological findings reported by Chan et al. [1,39] and a retina-specific conditional *VHL*-knockout mouse model by Kurihara et al, which exhibited mice with poorly formed retinal vessels, particularly in the peripheral retina [40]. However, the underlying mechanism influencing this location preference remains incompletely understood. 

In typical RHs caused by *VHL* mutations, the degradation of hypoxia-inducible factor (HIF) is reduced, leading to the signaling of hypoxia and subsequent upregulation of hypoxia-induced genes, such as the vascular endothelial growth factor (VEGF), erythropoietin (Epo), platelet-derived growth factor (PDGF-β) and transforming growth factor (TGF-α) [1]. It is worth noting that peripheral retina perfusion is often compromised in ischemic retinal diseases [41,42]. In the context of the VHL syndrome, biallelic VHL inactivation in cells is thought to mimic the effect of hypoxia [16]. Consequently, we propose that the peripheral retina, nourished by the distal ends of retinal vessels, may be more prone to hypoxia and thus more susceptible to angiogenesis. 

Our series is consistent with the conclusions reached by Weiley et al. [12] and Dalvin et al. [2], who contended that eyes with juxtapapillary and large extrapapillary RHs pose a difficult challenge in treatment. Recently, Belzutifan, a first-in-class HIF inhibitor, has been approved by the FDA for VHL-associated tumors [43,44,45], and it showed preliminarily promising effects on RHs [46,47]. There is hope that such pharmacotherapies might offer an alternative to the presently used destructive therapies.

## 5. Limitations

Certain limitations of our study include a restricted sample size, limiting our understanding of the full scope of the phenotype–genotype correlation. Moreover, while we focused on the RHs, not all patients underwent systemic examinations for other organ abnormalities due to poor compliance, introducing potential bias. The variability in patient follow-up duration could also result in underestimation of the progressive, multi-system involvements of the disease. Additionally, since our study was initiated as early as the year 2006, the genetic diagnosis of almost all patients in our series relied on Sanger sequencing and UPQFM-PCR rather than the next-generation sequencing widely applied nowadays; therefore, the changes in proximity of *VHL* in large deletions and the modifier genes were not sequenced. Despite these limitations, our study encompasses a significant cohort of the Chinese population, providing valuable insights into the clinical and genetic features of the disease.

## 6. Conclusions

In conclusion, our research characterizes the phenotypic and genotypic features of one of the largest series of RH in the Chinese population. The baseline age at which individuals with VHL-associated RH visit the hospital is significantly lower compared to non-VHL patients. We identified four novel variants of the *VHL* gene and determined codons 167, 161 and 86 to be the hotspots of mutation in VHL. Large deletions that result in the failure of protein production were observed at a notably high frequency in our series. Our findings highlight a predilection of the peripheral retina of tumor occurrence and presume that higher susceptibility to hypoxia of the peripheral retina might be a potential cause. Furthermore, our study reveals a strong association between hemangioblastoma in the CNS and the retina in patients with VHL-associated RH, implying that RH could potentially serve as an early indicator of CNS hemangioblastoma.

## Figures and Tables

**Table 2:** Variants identified in the *VHL* gene.

Variant Type	Location	cDNA Change	Protein Change	Domain	ALIGN GVGD	Polyphen2	Mutation Taster	ACMG * ^	In Silico	Cosegregation	Novelty	Nbr of Cases
**Point**	Exon 1	c.194C>G	p.Ser65Trp	β	176.58	Probably damaging	Deleterious	PS1	C65	AD		2
	Exon 1	c.194C>T	p.Ser65Leu	β	144.08	Possibly damaging	Deleterious	PS1	C65	AD		1
	Exon 1	c.208G>A	p.Glu70Lys	β	56.87	Benign	Deleterious	PS1	C55	Sporadic		1
	Exon 1	c.233A>G	p.Asn78Ser	β	46.24	Probably damaging	Deleterious	PS1	C45	AD		1
	Exon 1	c.233A>T	p.Asn78IIe	β	148.91	Probably damaging	Deleterious	PS1	C65	AD		1
	Exon 1	c.236G>C	p.Arg79Pro	β	102.71	Probably damaging	Deleterious	PS1	C65	AD		1
	Exon 1	c.256C>A	p.Pro86Thr	β	37.56	Probably damaging	Deleterious	PS1	C35	Sporadic		1
	Exon 1	c.256C>T	p.Pro86Ser	β	73.35	Probably damaging	Deleterious	PS1	C65	AD		1
	Exon 1	c.266T>C	p.Leu89Pro	β	97.78	Probably damaging	Deleterious	PS1	C65	AD		1
	Exon 1	c.278G>A	p.Gly93Asp	β	93.77	Probably damaging	Deleterious	PS1	C65	Sporadic		1
	Exon 1	c.335A>C	p.Tyr112Ser	β	143.11	Probably damaging	Deleterious	PM1, PP2, PM2, PM5, PP3, PP5	C65	AD	Novel	1
	Exon 1	c.349T>G	p.Trp117Gly	β	183.79	Possibly damaging	Deleterious	PS1	C65	AD		1
	Exon 1	c.393C>A	p.Asn131Lys	β	93.88	Probably damaging	Deleterious	PS1	C65	Sporadic		1
	Exon 3	c.470C>T	p.Thr157IIe	α	89.28	Probably damaging	Deleterious	PS1	C65	AD		1
	Exon 3	c.475A>G	p.Lys159Glu	α	56.87	Probably damaging	Deleterious	PS1	C55	AD		1
	Exon 3	c.499C>T	p.Arg167Trp	α	101.29	Probably damaging	Deleterious	PS1	C65	2 AD, 1 sporadic		3
	Exon 3	c.500G>A	p.Arg167Gln	α	42.81	Probably damaging	Deleterious	PS1	C35	Sporadic		1
**In-frame deletion**	Exon 3	c.528_530delGAG	p.176delArg	α	-		Deleterious	PM2, PM4, PM1,	-	AD	Novel	1
**Frameshift/Nonsense**	Exon 1	c.254insT	p.Leu85Leufs*46	β	-		Deleterious	PVS1, PM2,	-	AD	Novel	1
	Exon 1	c.294C>G	p.Tyr98*	β	-		Deleterious	PS1	-	AD		1
	Exon 1	c.329Adel	p.His110fs*49	β	-		Deleterious	PVS1, PM2,	-	AD		1
	Exon 3	c.481C>T	p.Arg161*	α	-	Probably damaging	Deleterious	PS1	-	1 AD, 1 sporadic		2
	Exon 3	c.515delCinsGCT	p.Pro172Argfs*30	α	-		Deleterious	PVS1, PM2,	-	AD	Novel	1
**Splicing**	Intron 2	c.463+3 A>G					Deleterious			AD		1
**Large deletions**	Exon 1							PVS1		AD		2
	Exon 2							PVS1		AD		1
	Exons 2 and 3							PVS1		AD		3

GVGD: Grantham variation (GV) and Grantham deviation (GD); * ACMG-AMP (American College of Medical Genetics and Genomics–American Association of Molecular Pathology) 2015 sequence (5821) or 2020 ACMG-ClinGen CNV (5822) guidelines. All known variants have the criteria shown, as well as PP1, PP2, PP3 and PP4; AD: autosomal dominant; ^ Franklin by Genoox (https://franklin.genoox.com/clinical-db/home, accessed on 1 January 2023) rated all novel variants as likely pathogenic; reference sequences: NM_000551.4, NP_000542.

**Table 3:** Complications of extrapapillary RHs.

	Largest RH Diameter	≤1.5 mm (*n* = 11)		1.5–4.5 mm (*n* = 24)		≥4.5 mm (*n* = 18)		Total (*n* = 53)		*p* (<0.013 Corrected)
		Nbr (Eyes)	Percentage	Nbr (Eyes)	Percentage	Nbr (Eyes)	Percentage	Nbr (Eyes)	Percentage	
**Retinal Complications (Baseline)**	ERD	0_a_	0%	3_a_	12.50%	14_b_	77.78%	17	32.08%	<0.001
	TRD	0	0%	4	16.67%	3	16.67%	7	13.21%	NS
	PVR	0	0%	5	20.83%	4	22.22%	9	16.98%	NS
	RRD	0	0%	1	4.17%	1	5.56%	2	3.77%	NS
**Macular Complications (Baseline)**	Macular involvement	0_a_	0%	14_b_	58.33%	16_b_	88.89%	30	56.60%	<0.001
	ME	0	0%	7	29.17%	3	16.67%	10	18.87%	NS
	ERM	0	0%	2	8.33%	3	16.67%	5	9.43%	NS
	Exudate	0	0%	5	20.83%	8	44.44%	13	24.53%	NS
	RD in macula	0	0%	4	16.67%	6	33.33%	10	18.87%	NS
**Invasive Treatment (Follow-Up)**	RV without SO	0	0%	1	4.17%	1	5.56%	2	3.77%	NS
	RV SO	2	18%	3	12.50%	7	38.89%	12	22.64%	NS
**Final Visit Status**	SO Removed	1	9%	1	4.17%	5	27.78%	7	13.21%	NS
	Long-term SO tamponade	1	9%	2	8.33%	2	11.11%	5	9.43%	NS
	NVG/NLP	0	0%	2	8.33%	3	16.67%	5	9.43%	NS

Each subscript letter denotes a subset of size categories, whose column proportions do not differ significantly from each other at the 0.05 level. ERD: exudative retinal detachment; TRD: tractional retinal detachment; PVR: proliferative vitreoretinopathy; RRD: rhegmatogenous retinal detachment; ME: macular edema; RD: retinal detachment; RV: vitreoretinal surgery; SO: silicone oil tamponade; NVG: neovascular glaucoma; NLP: no light perception.

## Data Availability

No new data were created or analyzed in this study. Data sharing is not applicable to this article.

## Supplementary Materials

The following supporting information can be downloaded at: https://www.mdpi.com/article/10.3390/genes15091192/s1.
